# Influence of different methods for calculating gestational age at birth on prematurity and small for gestational age proportions: a systematic review with meta-analysis

**DOI:** 10.1186/s12884-023-05411-0

**Published:** 2023-02-11

**Authors:** Gabriela Luiza Nogueira Vitral, Roberta Maia de Castro Romanelli, Tiago Alves Leonel, Juliano de Souza Gaspar, Regina Amélia Lopes Pessoa de Aguiar, Zilma Silveira Nogueira Reis

**Affiliations:** 1 Faculdade de Medicina, Universidade Federal de Minas Gerais, 30.130.100, Belo Horizonte, Avenida Professor Alfredo Balena, 190, Sala 601 Brazil; 2 Faculdade de Ciências Médicas de Minas Gerais, Alameda Ezequiel Dias, 275, Belo Horizonte, 30130-110 Brazil

**Keywords:** Gestational age, Prematurity, Ultrasound, Last menstrual period

## Abstract

**Background:**

Recognizing premature newborns and small-for-gestational-age (SGA) is essential for providing care and supporting public policies. This systematic review aims to identify the influence of the last menstrual period (LMP) compared to ultrasonography (USG) before 24 weeks of gestation references on prematurity and SGA proportions at birth.

**Methods:**

Systematic review with meta-analysis followed the recommendations of the PRISMA Statement. PubMed, BVS, LILACS, Scopus-Elsevier, Embase-Elsevier, and Web-of-Science were searched (10–30-2022). The research question was: (P) newborns, (E) USG for estimating GA, (C) LMP for estimating GA, and (O) prematurity and SGA rates for both methods. Independent reviewers screened the articles and extracted the absolute number of preterm and SGA infants, reference standards, design, countries, and bias. Prematurity was birth before 37 weeks of gestation, and SGA was the birth weight below the p10 on the growth curve. The quality of the studies was assessed using the New-Castle-Ottawa Scale. The difference between proportions estimated the size effect in a meta-analysis of prevalence.

**Results:**

Among the 642 articles, 20 were included for data extraction and synthesis. The prematurity proportions ranged from 1.8 to 33.6% by USG and varied from 3.4 to 16.5% by the LMP. The pooled risk difference of prematurity proportions revealed an overestimation of the preterm birth of 2% in favor of LMP, with low certainty: 0.02 (95%CI: 0.01 to 0.03); I^2^ 97%). Subgroup analysis of USG biometry (eight articles) showed homogeneity for a null risk difference between prematurity proportions when crown-rump length was the reference: 0.00 (95%CI: -0.001 to 0.000; I^2^: 0%); for biparietal diameter, risk difference was 0.00 (95%CI: -0.001 to 0.000; I^2^: 41%). Only one report showed the SGA proportions of 32% by the USG and 38% by the LMP.

**Conclusions:**

LMP-based GA, compared to a USG reference, has little or no effect on prematurity proportions considering the high heterogeneity among studies. Few data (one study) remained unclear the influence of such references on SGA proportions. Results reinforced the importance of qualified GA to mitigate the impact on perinatal statistics.

**Trial registration:**

Registration number PROSPERO: CRD42020184646.

**Supplementary Information:**

The online version contains supplementary material available at 10.1186/s12884-023-05411-0.

## Background

A qualified gestational age (GA) is essential to support healthcare decisions and to guarantee reliable perinatal health indicators for planning public policies [1]. The difference between the date of birth and the last menstrual period (LMP) is a useful method for GA calculation. However, the evaluation of GA by ultrasonography (USG) in early pregnancy with the crow-rump length (CRL) assessment is currently the most accurate method for dating pregnancy [2]. Clinical dating by LMP is fairly easy information to obtain GA in a birth scenario, however not always accurate being subject to memory bias, irregular menstrual cycles, and breastfeeding, among others [3]. Even easy to access, birth weight, as a single data, is not enough to discriminate preterm from term or to identify SGA newborns, although it is a risk marker for newborns [4]. The birth weight, gender, and GA allow the identification of the small for GA (SGA) newborn, according to weight below the 10th percentile for the standard expected in the growth curve [5].

Prematurity and nutritional classification at birth depend on the GA, which is essential to evaluate neonatal risks since they are associated with the chance of adverse neonatal outcomes [6]. Prematurity is the leading cause of death in children under five years old and is responsible for one million neonatal deaths annually [7, 8]. Recognizing the premature newborn, SGA, or both conditions at birth is important for the care provided at birth, as it helps timely interventions, indicating actions for greater effectiveness in care [9, 10]. In addition, the absence of a reliable gestational chronology negatively impacts the correct use of fetal and neonatal growth curves and leads to inaccuracy in epidemiological information associated with birth conditions [11, 12]. Affecting perinatal statistics, the scarcity of healthcare funding is still one determinant of the low access to prenatal USG in low-income countries (LMIC). This contributes to poor prenatal care coverage and makes estimating GA reliability even more difficult [1, 13]. The evaluation of GA by USG in early pregnancy with the crow-rump length (CRL) assessment is currently the most accurate method for redating pregnancy, but it requires high-cost equipment, early prenatal care, and a specialized health professional [2].

It is still unknown how much LMP and USG dating affect the rates of prematurity and SGA rates, based on studies with a high level of scientific evidence. Each method of gestational calculation can interfere differently with these rates. Previous publications point to the possibility that the LMP overestimates the rate of premature and post-term births, whereas the more advanced obstetric USG performed in GA could underestimate such dating [14, 15]. In this context of uncertainties, this systematic review aims to identify the influence between LMP compared with USG before 24 weeks of gestational references on prematurity and SGA rates at birth.

## Methods

The research protocol followed the recommendations of the PRISMA Statement [16] and was registered in the International Prospective Registry of Systematic Reviews under PROSPERO number: CRD42020184646. This systematic review included all publications available in bibliographic bases: PubMed (MEDLINE), Scopus-Elsevier, Embase-Elsevier, BVS/LILACS, and Web of Science, until the date of October 2022. The research question considered “PECO” structuring: (P) newborns, (E) ultrasound for estimating GA, (C) LMP for estimating GA and (O) prematurity and SGA rates for both methods. The complete search strategy, adopting specific descriptors linked to Boolean operators, was "(''gestational age" OR "pregnancy dating") AND ("last menstrual period" OR "menstrual date") AND (ultrasound OR ultrasonography OR "diagnostic imaging" OR ultrasonic) AND ((premature OR Preterm OR "immaturity at birth" OR prematurity) OR (small for gestational age" OR SGA OR "low birth weight" OR “intrauterine growth restriction” OR “small birth size”))". The search strategy was applied for descriptors present in the title, abstract, and keywords.

### Study eligibility criteria

The inclusion criteria were: [1] to have the GA at birth calculated by LMP and obstetric USG up to 24 weeks of gestation; [2] to have information about the data source for calculating the GA; [3] to have specific information about the standard fetal growth curve used to diagnose SGA. Primary studies, cohorts, epidemiological analyses, and database studies were also considered. There were no restrictions on language and date of publication. The exclusion criteria were: [1] GA at birth estimated by combining the two methods, [2] study with subgroups of newborns presenting specific diseases or conditions, such as carriers of congenital anomalies or growth anomalies, [3] population or sample composed only of premature or only SGA.

The preterm birth proportions was defined as births before 37 weeks of gestation divided by the total number of births. SGA was birth weight below the 10th percentile for gestational age and gender based on the growth chart.

### Study appraisal and synthesis methods

We used the State of the Art software through Systematic Review (StArt) [17] to import articles and to support identifying duplicates, exclusions, and inclusions. Independent reviewers screened the title and abstract and performed the full reading with extracted the absolute number of preterm infants and SGA, reference standards, design, and countries. This research had two pairs of independent reviewers for each step, and a third reviewer was the judge in case of disagreements. According to the registered protocol, the absolute and relative values of the number of preterm infants and SGA were extracted, in addition to the characteristics of the primary studies,.

### Evaluation of the quality of studies

Two independent reviewers assessed the methodological quality of the studies using the Newcastle–Ottawa Scale (NOS) [18] adjusted for the context of this review, detailed in Additional file 1. In cohort studies, 10 stars were possible, four for sample selection, two for comparability between the two GA estimation techniques, and four for outcomes. For cross-sectional studies, nine stars were possible, in which the outcomes part was scored with a maximum of three stars, valuing the appropriate and clearly described statistical treatment. So, lower-quality articles obtained fewer stars.

### Statistical analysis

The difference between two proportions using the risk difference [19] compared preterms when using LMP-based GA and prenatal USG-based GA. When the information was found, the same approach was used for the newborn SGA proportions. The null difference between prematurity proportions was adjusted in the center on the forest-plot graphs, with 95% confidence intervals (95%CI), considering two decimal places, according to Revman default. In this way, a result of 0.00 does not necessarily means zero difference, but the third decimal place onwards might have been hidden. Values below zero corresponded to a greater number of newborns being classified as preterm in favor of USG-based GA, while values above zero indicated greater proportions of preterm birth classification in favor of LMP-based GA. The random-effects model was adopted to mitigate high heterogeneity. The heterogeneity among studies was calculated using Chi^2^ and I^2^ for inconsistencies among proportions.

### Subgroup and meta-regression analysis

Subgroups were analyzed by study design: cohort or cross-sectional, income countries’ economies: LMIC or high-income countries (HIC), and antenatal USG measurements: crown rump length (CRL) or biparietal diameter (BPD) biometrics. The Review Manager software (RevMan 5.4.1) was used for the meta-analysis.

## Results

**Fig. 1:**
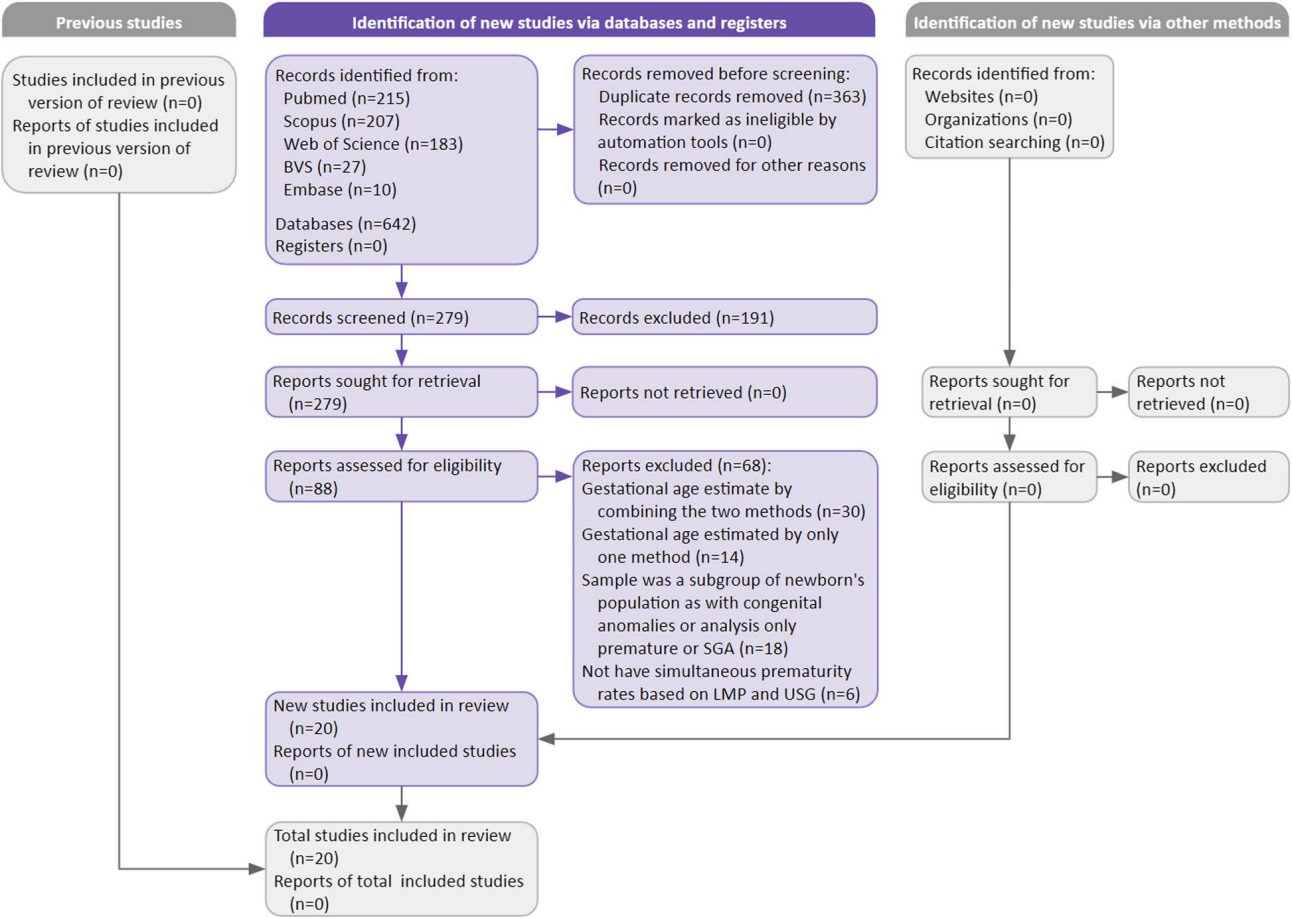
Study selection process. SGA: Small for gestational age, LMP: Date of last menstrual period, USG: Ultrasonography

The selection procedure of articles is shown in Fig. 1. In total, 642 articles were found, 215 PubMed, 207 Scopus, 183 Web of science, 27 BVS/Lilacs, and 10 Embase. Finally, 20 articles met the criteria for data extraction and synthesis according to the selection process depicted. Only one [15] among the 20 articles evaluated the SGA proportions estimate considering the 2 methods (LMP and USG).

We detailed the general characterization of the articles in Table 1. Among the 20 articles included in the meta-analysis, five presented a cross-sectional study design, and 15 were cohort studies. The year of publication ranged from 1995 to 2022, and the sample ranged from 171 to 165,908 newborns. Regarding the income countries of study location, ten were carried out in LMIC, such as Bangladesh, Colombia, Guatemala, Brazil, India, and Zambia, and ten in HIC, such as the USA, England, Denmark, and the Netherlands. Regarding the target population, eleven were carried out with mothers from the general population, two in rural areas, and seven were associated with other screening programs conducted widely in the studied population, such as the study of the Alpha-Fetoprotein Screening Program (XAPF) [20]. One report comparing pregnancy outcomes between women living with HIV and HIV-negative had data extraction only from the control group [21].

**Table 1:** General characterization of the articles eligible for the systematic review

Author	Year	Study design	N	Study location	Period	Target population
AMANHI Study group [22]	2022	Cohort	9,974	South-Asia and sub-Saharan Africa	Jul 2012 – Sept 2016	AMANHI participants group
Dietz, P. M., et al. [20]	2007	Cross-sectional	165,908	California, USA	2002	XAPF study participants
Gardosi, J.; Francis, A. [23]	2000	Transversal	21,069	Birmingham, England	1988—1995	General population
Gernand, A. D., et al. [24]	2016	Cohort	353	Bangladesh	Fev 2009—Mar 2010	Rural area
Gonzalez, L. G., et al. [25]	2015	Cross-sectional	344	Manizales, Colombia	Sept 2012	General population
Henriksen, T. B., et al. [26]	1995	Cohort	3,606	Denmark	1989—1991	General population
Hoffman, C. S., et al. [27]	2008	Cohort	1,867	North Carolina and Texas, USA	2000—2004	RFTS Study Participants
Malaba, T. R., et al. [21]	2021	Cohort	2,507	Cape Town, South Africa	Apr 2015 – Oct 2016	General Population
Medeiros, M. N. L., et al. [28]	2015	Cohort	2,847	Ribeirão Preto and São Luis, Brazil	Mar 2010—Out 2011	General Population
Mongelli, M.; Gardosi, J. [29]	1997	Cross-sectional	34,249	Nottingham, England	-	General Population
Naslund Thagaard, I., et al. [30]	2016	Cohort	8,551	Copenhagen, Denmark	2006—2012	General Population
Neufeld, L. M., et al. [31]	2006	Cohort	171	Rural Guatemala	Aug 1996—Jun 1999	Rural Guatemala
Nguyen, T. H., et al. [32]	1999	Cohort	17,450	Copenhagen, Denmark	Jan 1986—Dec 1996	General Population
Pereira, A.P., et al. [33]	2013	Cohort	1,483	Rio de Janeiro, Brazil	Dez 2007—Nov 2008	General Population
Price, J. T., et al. [14]	2019	Cohort	692	Lusaka, Zambia	Aug 2015—Aug 2017	ZAPPS Study Participants
Reuss, M. L., et al. [34]	1995	Cohort	367	Pennsylvania and New York, USA	Jan 1987—Jun 1989	General Population
Savitz, D. A., et al. [35]	2002	Cohort	3,655	North Carolina, USA	Out 1995—Mai 2001	PIN Study Participants
van Oppenraaij, R. H. F., et al. [36]	2015	Cross-sectional	24,665	Rotterdam, Netherlands	Jan 2000—Dec 2009	General Population
Vijayram, R. et al. [37]	2021	Cohort	1,721	India	Mai 2015 –Nov 2017	GARBH-Ini study participants (rural and semi urban)
Weinstein, J. R., et al. [15]	2018	Cohort	188	Guatemala	Mar 2013—Fev 2015	SPHB study participants

*N* number of participants in the study, *XAPF* Alpha-Fetoprotein Screening Program, *RFTS* Right from the Start, *ZAPPS* Zambia Prematurity Prevention Study, *PIN* Pregnancy Infection and Nutrition, *SPHB* Safe Pregnancy Healthy Baby, *GARBH-Ini* Interdisciplinary Group for Advanced Research on BirtH outcomes—India Initiative

Table 2 provides information on the data sources for estimating GA at birth. The moment of the pregnancy when assessment with the obstetric USG occurred and the source of this data. It covered the information source of the LMP, the proportions of prematurity, and SGA newborns by the two methods of estimate analyzed. Fourteen articles reported the USG report in the medical record, and the USG was performed by the researchers in six. Information about LMP was collected through maternal interviews in ten articles, and ten were searched in medical records. The prematurity proportion by USG ranged from 3.4 to 16.5% and from 1.8 to 33.6% by LMP. Only one study reported comparisons between the SGA proportions, which was 32% (95%CI: 25—39) by USG and 38% LMP (95%CI: 31—46) [15].

**Table 2:** Characterization of the studied methods LMP and US of the systematic review

									**Prematurity rates**			**SGA rates**	
**Author**	**Time of USG assessment**		**Source of USG data**	**Source of LMP data**		**LMP**	**USG**	**Difference**		**LMP**	**USG**		**Difference**
AMANHI [22]	8 to 19 weeks		Researcher exam	Maternal interview		21.6%*	9.8%*	11.8%		-	-		-
Dietz, P. M. et al. [20]	< 20 weeks		Medical record	Medical record		8.7%	7.9%	0.8%		-	-		-
Gardosi, J. and Francis, A. [23]	14 to 21 weeks		Medical record	Medical record		6.3%	7%	-0.7%		-	-		-
Gernand, A. D. et al. [24]	< 15 weeks		Researcher exam	Maternal interview		10.8%	8.7%	2.1%		-	-		-
Gonzalez, L. G. et al.[25]	56 to 97 days		Medical record	Medical record		5.2%	4.3%	0.9%		-	-		-
Henriksen, T. B., et al.[26]	< 20 weeks		Medical record	Medical record		4.0%	3.4%	0.6%		-	-		-
Hoffman, C. S., et al.[27]	4 to 17 weeks		Researcher exam	Maternal interview		10%	8.9%	1.1%		-	-		-
Malaba, T. R., et al. [21]	< 24 weeks		Researcher exam	Maternal interview		33.6%	14.0%	19.6%		-	-		-
Medeiros, M. N. L., et al.[28]	< 20 weeks		Medical record	Maternal interview		9.7%	8.5%	1.2%		-	-		-
Mongelli, M.; Gardosi, J.[29]	< 24 weeks		Medical record	Medical record		6.2%	6.6%	-0.4%		-	-		-
Naslund Thagaard, I., et al. (a) [30]	CRL between 45 and 84 mm		Medical record	Medical record		4.1%	4.5%	-0.4%		-	-		-
Naslund Thagaard, I., et al. (b) [30]	BPD first trimester		Medical record	-		4.1%	4.6%	-0.5%		-	-		-
Neufeld, L. M., et al.[31]	15 to 24 weeks		Researcher exam	Maternal interview		1.8%	4.1%	-2.3%		-	-		-
Nguyen, T. H., et al.[32]	12 to 22 weeks		Medical record	Medical record		4.0%	4.5%	-0.5%		-	-		-
Pereira, A. P., et al.[33]	7 to 20 weeks		Medical record	Maternal interview		17.7%*	13.2%	4.5%		-	-		-
Price, J. T., et al.[14]	< 20 weeks		Medical record	Medical record		20.2%	11.4%	8.8%		-	-		-
Reuss, M. L., et al.[34]	< 18 weeks		Medical record	Maternal interview		7.6%	10.1%	-2.5%		-	-		-
Savitz, D. A., et al.[35]	< 18 weeks		Medical record	Medical record		12.2%	12.1%	0.1%		-	-		-
van Oppenraaij, R. H. F., et al.[36]	8 weeks 0 day to 11 weeks 6 days		Medical record	Medical record		8.3%	8.3%	0%		-	-		-
Vijayram, R. et al. (a) [37]	< 20 weeks Hadlock		Researcher exam	Maternal interview		14.0%	14.5%	-0.5%		-	-		-
Vijayram, R. et al. (b) [37]	< 20 week Robinson-Fleming		Researcher exam	-		14.0%	16.5%	-2.5%		-	-		-
Weinstein, J. R., et al. [15]	< 24 weeks		Medical record	Maternal interview		17.8%	16.0%	1.8%		38%	32%		6%

*LMP* Last menstrual period, *SGA* Small for gestational age, *USG* Ultrasonography, Naslund (a) CRL measurement, Naslund (b) BPD measurement in the first trimester, Vijayram (a) US Hadlock, Vijayram (b) US Robinson-Fleming formula

^*^Average of countries

### Quality analysis of included articles

Table 3 shows the evaluation of the quality of the articles and, according to the NOS Scale. Regarding the cohort studies, three of them reached 9/10 stars [21, 24, 27]. The other studies are cross-sectional, with a maximum score of 7/9 stars in only two articles [32, 37]. The quality of the results refers to the independent or blind assessment of the prematurity proportions by the two references, the clarity in obtaining the date of birth, and the connection to the data of the beginning of pregnancy. In addition, it relates to the number of follow-up losses in the cohort and cross-sectional studies. The bias in the comparability between the proportions obtained by USG in relation to that obtained by LMP was based on the control of the reliability of the LMP. For this, we considered the resources used to certify this information, such as regular cycles, absence of abortion, and close birth influences on the female cycles, and statistical analysis. In database studies, efforts to qualify information on LMP and USG were better scored. Six studies presented a high risk of bias [25, 28, 30, 31, 33, 35].

**Table 3:** Quality evaluation of the studies selected in the systematic review by the Newcastle–Ottawa Scale

Study	Type	Selection	Comparability	Outcome	Total
AMANHI [22]	Prospective cohort	★★★	★	★★★	7/10
Gernand, A. D.; et al. [24]	Prospective cohort	★★★★	★	★★★★	9/10
Henriksen, T. B. et al. [26]	Prospective cohort	★★★	★	★★	6/10
Hoffman, C. S., et al. [27]	Prospective cohort	★★★★	★★	★★★	9/10
Malaba, T. R., et al. [21]	Prospective cohort	★★★★	★	★★★★	9/10
Medeiros, M. N. L., et al. [28]	Prospective cohort	★★	-	★★	4/10
Naslund T., et al. [30]	Prospective cohort	★	★	★★	4/10
Neufeld, L. M. et al. [31]	Prospective cohort	★★★★	-	-	4/10
Pereira, A.P., et al. [33]	Prospective cohort	★★★	-	★	4/10
Price, J. T., et al. [14]	Prospective cohort	★★★★	★	★	6/10
Reuss, M. L., et al. [34]	Prospective cohort	★★★	★	★★★	7/10
Savitz, D. A., et al. [35]	Prospective cohort	★★★★	-	-	4/10
Van Oppenraaij, R. H. F., et al. [36]	Retrospective cohort	★★	★★	★	5/10
Weinstein, J. R., et al. [15]	Prospective cohort	★★★	★	★★	6/10
Vijayram, R. et al. [37]	Prospective cohort	★★★	★	★★★	7/10
Dietz, P. M., et al. [20]	Cross-sectional	★★★	★	★	5/9
Gardosi, J. & Francis, A. [23]	Cross-sectional	★★★★	★	★	6/9
Gonzalez, L. G., et al. [25]	Cross-sectional	★	-	★	2/9
Mongelli, M. & Gardosi, J. [29]	Cross-sectional	★★★★	★	★	6/9
Nguyen, T. H., et al. [32]	Cross-sectional	★★★	★★	★★	7/9

Among the 15 cohort studies, the ones with the highest quality scores were those that stood out for their exceptional control in the prospective collection of LMP data, with control of preconception cycles [21, 24] and those that used prospective records or a large representation of the population of pregnant women [22, 27]. Among the five cross-sectional studies, the criterion with the highest risk of bias was the Outcome-item, either due to excessive data loss or a specific statistical approach to compare the prematurity proportions obtained by the two benchmarks. Part of the studies mainly focused on the analysis by grouping birth weight [20, 29] or prematurity subgroups [23, 25, 32].

### Risk difference between two proportions

**Fig. 2:**
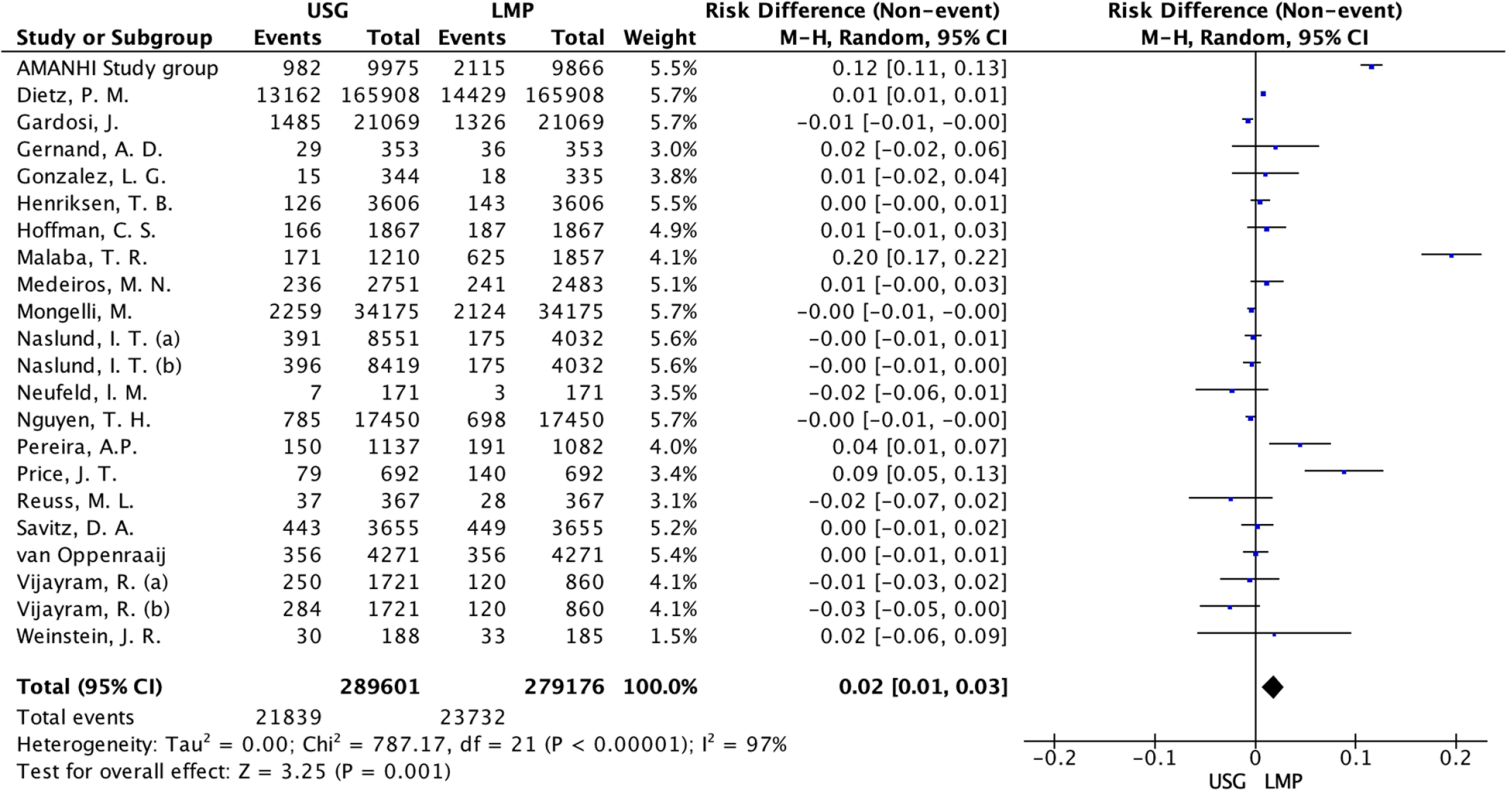
Forest plot of premature proportions by Last menstrual period and USG before 24 weeks. LMP: Last menstrual period; USG: Ultrasonography

Twenty-two proportions of prematurity were extracted from the 20 articles by each method, Fig. 2. The difference between preterm birth proportions was combined, resulting in 0.02 (95%CI: 0.01 to 0.03) in favor of LMP. It means that LMP-based GA overestimated the preterm birth proportions by 2%. However, the I^2^ value was 97%, *p* < 0.001, indicating high heterogeneity among the studies. In five studies, confidence intervals did not cross the null effect. These findings were robust since a sensitivity analysis removing the duplicity of proportions of prematurity [30, 37] showed a risk difference of 0.02 (95%CI: 0.01 to 0.03), I^2^ 98%,* p* < 0.001, Additional file 2.

**Fig. 3:**
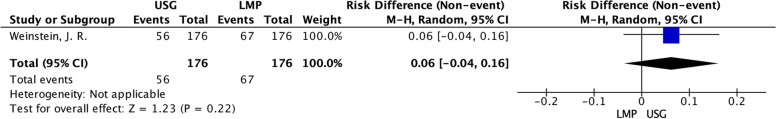
Forest plot of small-for-gestational-age proportions by last menstrual period and ultrasonography before 24 weeks. LMP: Last menstrual period; USG: Ultrasonography

Comparisons between SGA proportions were measured in one study. There was a non-significant effect of LMP or USG methods on the SGA proportion at birth, Fig. 3 since the risk difference was 0.06 (95%CI: -0.04 to 0.16), crossing the null value.

**Fig. 4:**
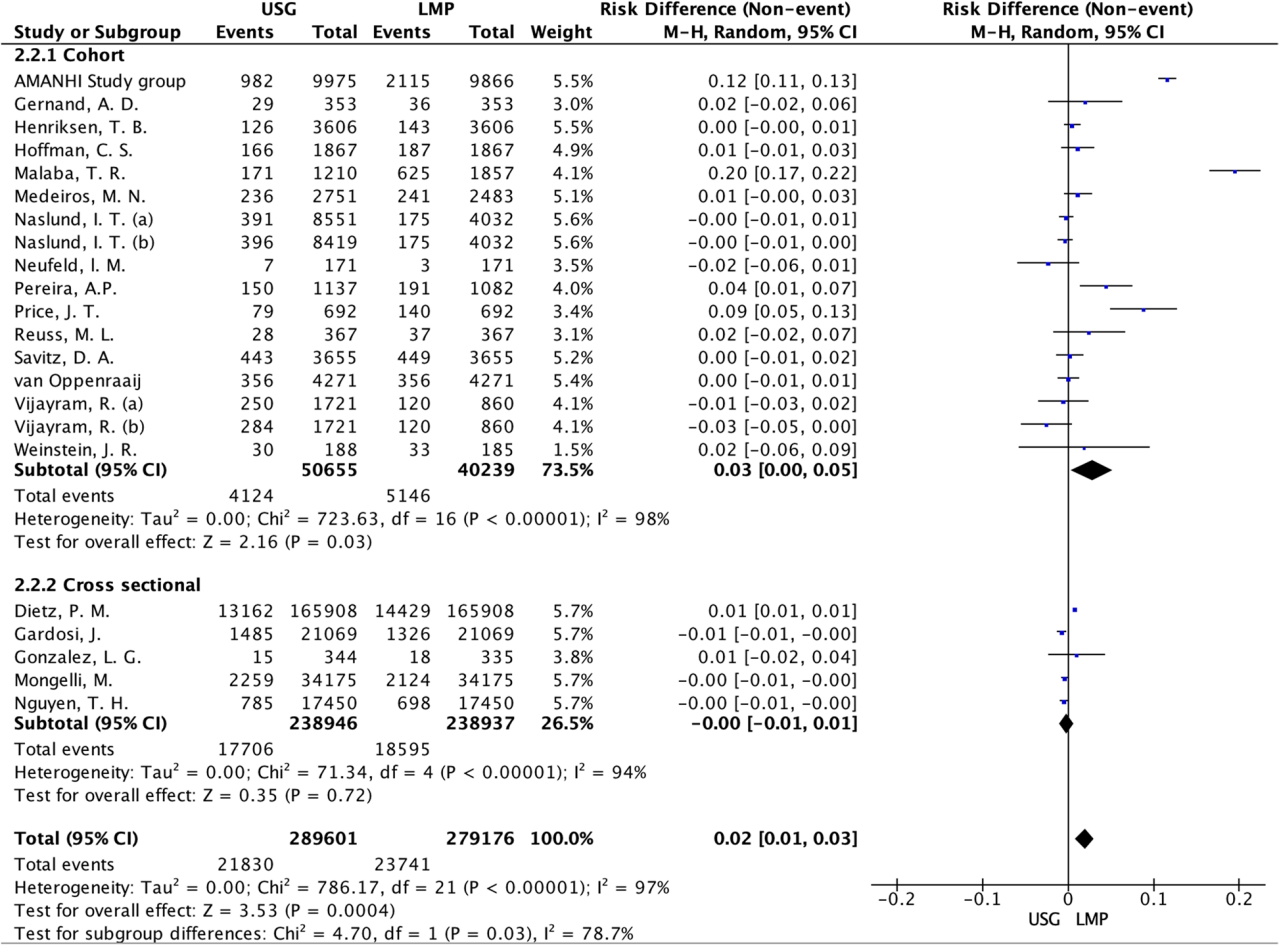
Forest plot of subgroup by study design: Cohort or cross-sectional. LMP: Last menstrual period; USG: Ultrasonography. Naslund (**a**): CRL measurement; Naslund (**b**): BPD measurement in the first trimester. Vijayram (**a**): US Hadlock; Vijayram (**b**): US Robinson-Fleming formula

In the subgroup analysis by study design Fig. 4, the pooled risk difference was 0.02 (95%CI: 0.01 to 0.03), I^2^ 97%, *p* < 0.001, with LMP-based GA overestimation preterm birth by 2%. Across the 15 cohorts, however, risk difference showed no effect between LMP or USG references on the preterm birth proportions, with substantial heterogeneity: 0.03 (95%CI: 0.00 to 0.05). Likewise, in the cross-sectional study subgroup, I^2^ 94%. These findings indicated that the study design may have unaffected the risk difference of prematurity since the total group and subgroups had high values of heterogeneity.

**Fig. 5:**
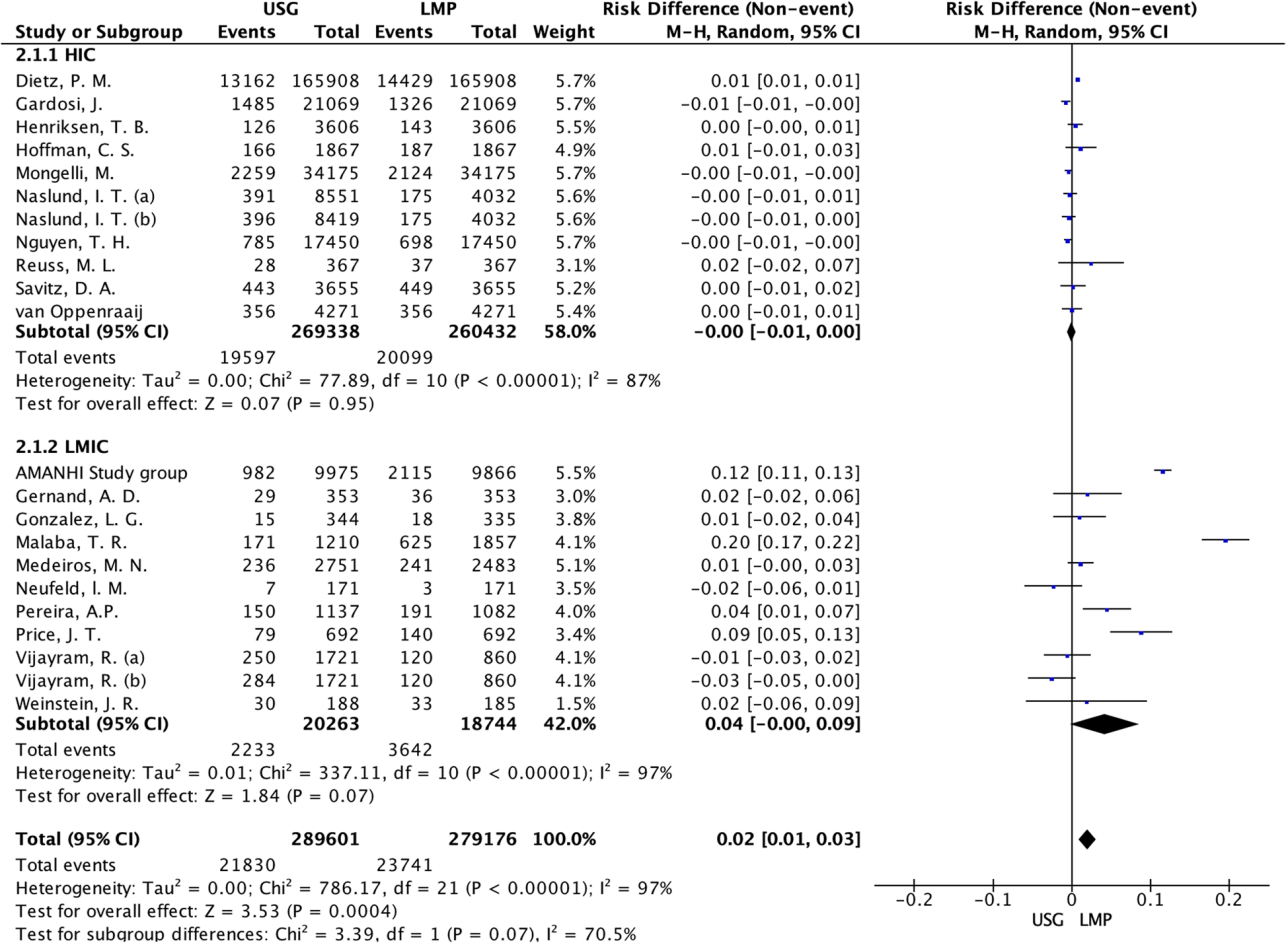
Forest plot of subgroup by country of study location: Low-middle Income countries or high-income countries. LMP: Last menstrual period; USG: Ultrasonography; HIC: high-income country; LMIC: Low-middle income countries. Naslund (**a**): CRL measurement; Naslund (b): BPD measurement in the first trimester. Vijayram (**a**): US Hadlock; Vijayram (**b**): US Robinson-Fleming formula

In the subgroup analysis by the country income of study Fig. 5, the risk difference of prematurity was 0.02 (95%CI: 0.01 to 0.03), I^2^ 97%, *p* < 0.001 in the studies conducted in LMIC. The findings indicated considerable heterogeneity among the studies, as well as in the subgroup of studies conducted in HIC in which I^2^ 87%, *p *< 0.001. The risk difference was not significant, 0.04 (95%CI: -0.00 to 0.09), I^2^ 97%, despite the higher prevalence of prematurity by LMP in countries of LMIC.

**Fig. 6:**
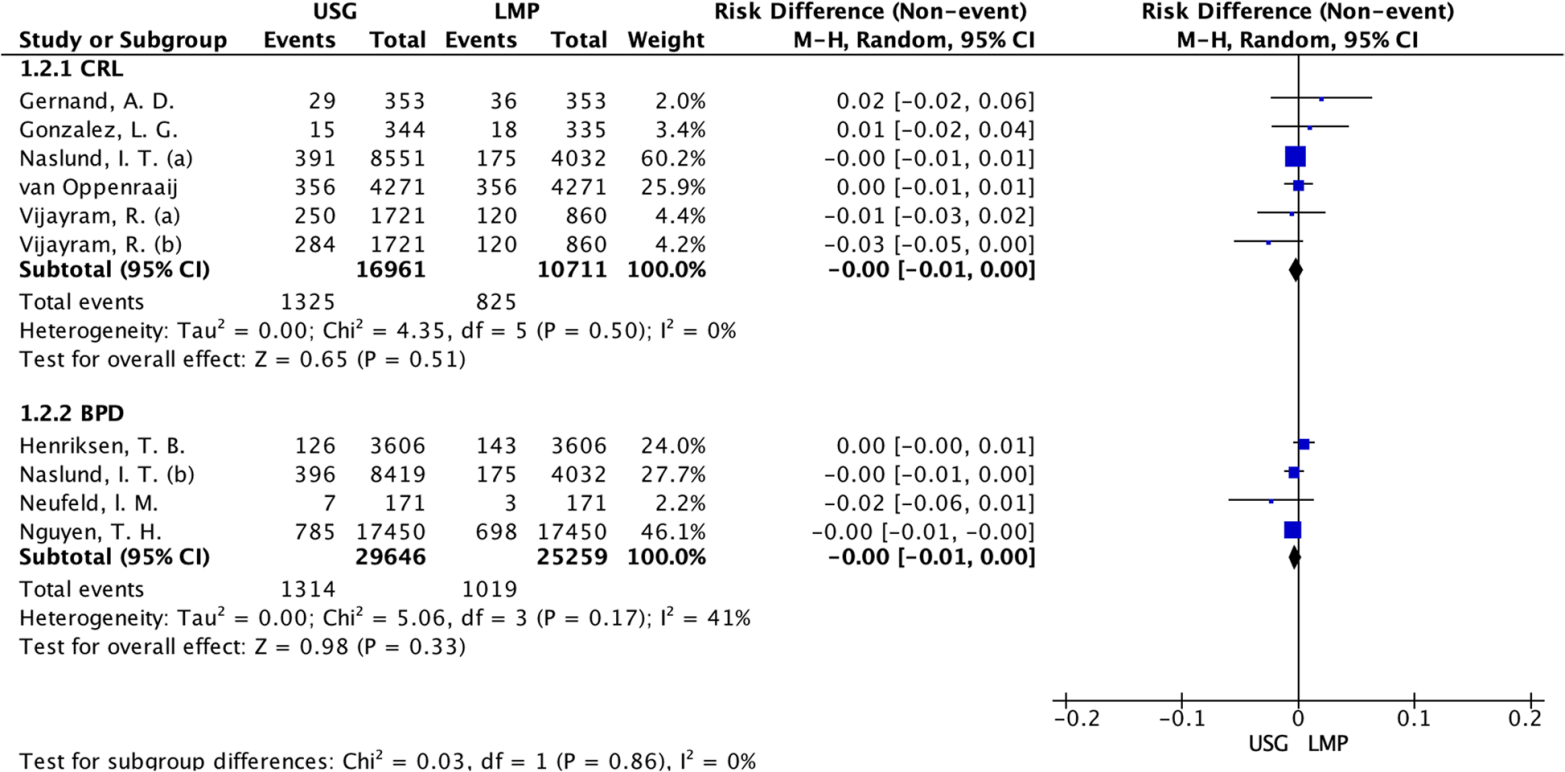
Forest plot of subgroup by antenatal USG measure: Crown-rump length or biparietal diameter. LMP: Last menstrual period; USG: Ultrasonography; CRL: Crown-rump length; BPD: Biparietal diameter. Naslund (**a**): CRL measurement; Naslund (**b**): BPD measurement in the first trimester. Vijayram (**a**): US Hadlock; Vijayram (**b**): US Robinson-Fleming formula

The USG captured CRL in five studies and BPD in four studies. In the subgroup analysis by antenatal USG measure Fig. 6, the risk difference for prematurity proportions was null calculated by CRL and null calculated by BPD when USG was used. Besides, we highlighted a fall in the heterogeneity among the studies for both methods of measure, considering I^2^ 0% (*p* = 0.50) in the CRL measure and I^2^ 41% (*p* = 0.17) in the BDP measure. It revealed the importance of segment of body measure by USG to explain the high heterogeneity among studies partially.

Thus, considering 20 reports, there was a low (2% in favor of LMP), however, with a high uncertainty risk difference in prematurity proportions between methods of reference. However, the CRL assessment in early pregnancy as a reference for GA resulted in similar prematurity proportions compared to the LMP reference.

## Discussion

This review compared the proportions of prematurity and SGA between LMP and USG before 24 weeks as a reference for estimating GA. The occurrence of preterm among infants varied significantly in the reports, with prematurity proportions ranging from 1.8% to 20.2% by LMP [14, 31]. It means different complexities of health assistance from varied countries and diverse ultrasonography approaches. In an attempt to reduce such differences, the meta-analysis considered the random effects model, sensitivity, and subgroup analysis. Five studies showed a significant risk difference for prematurity in favor of the GA calculated by the LMP [14, 20–22, 33], and no studies by the USG-based GA. In the all set of studies, a significant risk difference of 2% (1% to 3% 95%CI) was found between methods in favor of higher prematurity proportions when using LMP. Our interpretation regarding this outcome relies on the tendency for underestimation of GA based on LMP in preterm newborns and the overestimation in the term and post-term compared to GA based on USG. The overlapped GA distribution comparisons between LMP and USG references to support this possibility were represented in previous reports [22, 38]. We speculate such differences unbalance the proportions in the direction of higher prematurity. Even with high heterogeneity and moderate risk of bias, we raised concern about the overestimation of preterm rates in birth scenarios where GA is mainly calculated based on LMP. Regarding the comparison of SGA proportions, the lack of studies kept unclear the influence of such references for GA calculation on the indicator.

### Investigation of subgroup analyses

#### Income

The subgroup analysis supported that the difference between proportions of prematurity is practically null in the scenario of HIC since the pooled result had no evidence of prematurity in favor of any side. Early access to prenatal care and higher quality of assistance may be associated with this finding [39]. Studies in LMIC countries had risk differences between methods in favor of higher prematurity proportions when using LMP-based GA. However, it presented a 95% CI reaching the null value. Among the ten studies in this group, four showed evidence of significant risk differences in favor of LMP [14, 21, 22, 33], and two studies found differences in favor of USG, but not significant [31, 37]. Most of the studies in this subgroup presented high proportions of prematurity as compared to those in the HIC group. One possible interpretation is that the qualification of clinical information about menstrual cycles was more valuable in studies of LMIC, while the early access to prenatal care, including USG, may be limited [6].

#### Study design

Regarding the study's design, the group of articles with a cohort approach had a similar and high I^2^ value compared to the cross-sectional group of articles (98% vs. 94%, respectively). Thus, the study design did not explain the high heterogeneity among the studies. With cohort design, 15 articles gathering 50,655 newborns, a difference between prematurity proportions was in favor of LMP when compared to USG, with high uncertainty. Otherwise, in the cross-sectional study subgroup, five articles with 238,946 newborns, there was no evidence of a difference in favor of any side. Prospective cohorts of pregnant women occurred in scenarios with a high prematurity proportion in LMICs [14, 21, 22, 33], all articles in favor of LMP-based GA overestimating prematurity proportions. Also, attention is drawn to a large number of cases that Dietz et al. (2007) studied in a cross-sectional analysis of 165,908 women participating in the XAPF screening program in California, USA [20], also with a risk difference in favor of LMP-based. The other studies showed a 95% CI for the risk difference crossing the null effect, i.e., without evidence of impact on the proportions of prematurity.

#### Antenatal USG measure

Stratified analysis of USG biometry revealed high homogeneity among six-paired risk differences of five studies when CRL was the reference for GA estimate at birth [24, 25, 30, 36, 37]. Even though such pooled risk difference leaned to the USG-based GA side, there was no significant evidence that prematurity proportions based on CRL would differ from LMP reference. The CRL assessment in early pregnancy resulted in similar prematurity proportions compared to the LMP reference. The clinical application of this outcome is aligned with prior reports when the CRL is considered the most accurate method for GA redating [2]. Similarly, the risk difference of prematurity when DPB is available < 24 weeks was the reference for GA leaned to the USG side, however, with high uncertainty. Further comparative studies are still needed to confirm this trend. This last finding corroborates the USG before 24 weeks gestation as an acceptable standard the World Health Organization recommended to improve prenatal care [40]. For a proper interpretation, the inclusion criterion for USG < 24 weeks may have influenced the high heterogeneity among the set of studies since the fall of heterogeneity was clear using USG biometry subgroup analysis. CRL measurement is the best parameter for calculating the GA [2]. However, we consider that using first-trimester USG with CRL measurement would have limited the inclusion of many studies, especially those carried out in LMIC.

#### Quality of the studies

The main bias domain in the systematic review was the procedure to access the reference used in the GA calculation. It is worth mentioning that collecting information about the menstrual cycle varied among the studies. This information was obtained prospectively in rural Bangladesh [24], Guatemala [31], and others [14, 21] through the woman's self-report or from a medical record and database, most of them without mention of procedures for data qualification. Accordingly, the discrepancy in the proportion of prematurity found in the same country is noteworthy: in the study by Weinstein et al., 17.8% and Neufeld et al.,1.8% based on the LMP [15, 31]. Even though these studies analyze different birth scenarios, the figures are still far from Guatemala's prematurity rate, estimated at 10% [41]. The quality of the prematurity rate is directly linked to the accuracy of the GA estimate. Incompleteness, lost, and underreported data are more frequent limitations in LMICs [40]. Several factors are recognized for affecting the quality of data in this indicator, such as the collection protocols used by health professionals, early access to prenatal care and USG, and the value of this information for women, caregivers, and governments, among others [42].

#### Strength and limitations of the review

The main contribution was to provide unprecedented comparison proportions of prematurity and SGA by two references recommended for calculating GA. However, the results found present limitations regarding the variability of research designs, different USG biometrics, and contexts of birth scenarios among the reports, which would possibly explain the heterogeneity observed in the set of all articles, at least in part. The considerable heterogeneity in the meta-analysis was a limiting factor in interpreting the results for clinical practice. Future controlled studies should mitigate methodological weakness in blinding the GA between methods. Another point to highlight was the duplication of prematurity proportions by different USG approaches, Hadlock vs. Robinson-Fleming; and CRL vs. DBP. However, the sensitivity analysis remained similar to the total 22 prematurity proportions, considering just one per study.

Despite the limitations, this review is helpful as a basis for studies involving estimates of GA and its relationship to the prematurity and SGA proportions. Transparent criteria in the study group selection, appropriate statistical treatment, and a clearly described and detailed methodology are essential for a study to confirm the differences among the rates of prematurity by the two reference methods. Another point was the lack of studies comparing the SGA proportions obtained by different reference calculations for GA.

The correct determination of GA can affect the results of pregnancy [43], improve decision-making in childbirth and neonatal care [44], optimizing health costs [45]. Investigating the influence of different references for prematurity and SGA rates is relevant for any delivery scenarios and public policies and research [13, 46, 47]. A valid prematurity rate is unknown in many places due to the lack of qualified data, especially in LMIC [47]. This systematic review reinforced the importance of early prenatal care with qualified LMP and USG access to adjust the due date of birth. The lack of studies kept unclear the influence of LMP and USG references on SGA proportions. Insights for future primary research are to compare the rates paying attention to the fetal biometric measures for pregnancy dating and considering the diversity of high-cost healthcare technologies access.

## Conclusions

Meta-analysis showed that LMP compared to the USG before 24 weeks of gestational references for GA calculation, has little or no effect on prematurity proportions at birth, considering the high heterogeneity among studies. The CRL by USG assessment in early pregnancy resulted in similar prematurity proportions compared to the LMP reference. The lack of studies kept unclear the influence of such references on SGA proportions. Results corroborated the importance of qualified GA to mitigate the impact on perinatal statistics.

## Supplementary Information

**Additional file 1.** NewCastle-Ottawa scale adjusted for the context of the review

**Additional file 2.** Forest plot of premature proportions by last menstrual period and USG before 24 weeks without duplicated proportions

## Data Availability

The datasets used and/or analyzed during the current study are available from the corresponding author on reasonable request.

## Abbreviations

BPD: Biparietal diameter
CRL: Crown-rump length
GA: Gestational age
HIC: High-Income Countries
LMIC: Low-Middle Income Countries
LMP: Date of last menstrual period
NOS: New Castle-Ottawa scale
SGA: Small for gestational age
USG: Ultrasonography
