# Supplementary material for: Influence of different methods for calculating gestational age at birth on prematurity and small for gestational age proportions: a systematic review with meta-analysis
Source: BMC Pregnancy Childbirth. 2023 Feb 11;23:106. doi: 10.1186/s12884-023-05411-0 (PMC9921121; doi:10.1186/s12884-023-05411-0)
Supplement: Supplementary file 1 — Additional file 1. NewCastle-Ottawa scale adjusted for the context of the review [file 12884_2023_5411_MOESM1_ESM.docx]

**Additional file 1.** NewCastle-Ottawa scale adjusted for the context of the review

NEWCASTLE - OTTAWA QUALITY ASSESSMENT SCALE

COHORT STUDIES

Note: A study can be awarded a maximum of one star for each numbered item within the Selection and Outcome categories. A maximum of two stars can be given for Comparability

***Selection (4 stars):***

1) Representativeness of the exposed cohort

a) ★ Truly representative of pregnant women and newborns in the community: population cohort.

b) ★ Somewhat representative of pregnant women and newborns in the community: multicenter study, clinical trial derivation of the cohort or random sample.

c) Zero: sample from tertiary referral centers, selected groups of study, convenience sample. d) Zero: No description of the derivation of the cohort.

2) Selection of the no exposed to the early-antenatal-ultrasound, in the cohort a) ★ Drawn from the same community as the exposed cohort: eligibility criteria of inclusion based on the last menstrual period and early-ultrasound recollection were confirmed by the agreement of the total numbers in the results.

b) Zero: Drawn from a different source and eligibility criteria of inclusion of last menstrual period or early-ultrasound did not confirm by the agreement of the total numbers in the results.

c) Zero: no description of the derivation of the non-exposed cohort

3) Ascertainment of exposure to the early-antenatal-ultrasound, in the cohort a) ★ Secure record of last menstrual period with prospective register and controlled antenatal ultrasound assessment by the researchers

b) ★ Secure record of last menstrual period with quality control and early-ultrasound adjusted to a standard curve of fetal growth.

c) Zero: Self report of last menstrual period recollected at birth or no description of data source.

1

4) Demonstration that outcome of interest was not present at start of study a) ★ Yes

b) Zero: no

***Comparability (2 stars):***

5) Comparability of cohorts on the basis of the design or analysis

a) ★ Study controls for the last menstrual period data using qualified information of menstrual cycles.

b) ★ Study controls for the premature proportion and small for gestational age proportion differences between the early-ultrasound and last menstrual period references for gestational age, using statistics.

c) Zero: There was no comparison for the premature proportion and small for gestational age proportion between the early-ultrasound and last menstrual period references for gestational age, using statistics.

***Outcome (4 stars):***

6) Assessment of outcome

a) ★ Independent blind assessment.

b) ★ Record linkage of data.

c) Zero: no description.

7) Adequacy of follow up of cohorts

a) ★ Complete follow up - all enrolled subjects accounted at the birth setting with lost <25% (fetal death and abortion)

b) ★ Data missing unlikely to introduce bias - small number lost <=10 % (missing data with cohort fluxogram, or description provided of those lost).

c) Zero: Follow up proportion >25% or data missing >10%.

d) Zero: No statement.

2

NEWCASTLE - OTTAWA QUALITY ASSESSMENT SCALE

(adapted for cross sectional studies)

***Selection (4 stars):***

1) Representativeness of the sample

a) ★ Truly representative of pregnant women and newborns in the community: population database.

b) ★ Somewhat representative of of pregnant women and newborns in the community: all subjects or random sampling.

c) Zero: tertiary referral centers, selected groups of study, convenience sample. d) Zero: No description of the sampling.

2) Sample size

a) ★ Justified and satisfactory.

b) Zero: Not justified.

3) Selection of target population

a) ★ Comparability between gestational age based on the last menstrual period and early ultrasound confirmed by the agreement of the exposed and no-exposed numbers in the results.

b) Zero: Comparability between gestational age based on the last menstrual period and early ultrasound is unsatisfactory, according to the lack of the agreement of the total numbers in the results.

c) Zero: no description.

4) Ascertainment of exposure

a) ★ Secure record of last menstrual period recollected during early pregnancy and early ultrasound accessed by the researchers in medical records.

b) ★ Secure record of last menstrual period with quality control and early-ultrasound antenatal ultrasound adjusted to a standard curve of fetal growth.

c) Zero: Population database without data curation.

d) Zero: Self report of last menstrual period at birth or no description of data source.

3

***Comparability (2 stars):***

5) Comparability of cohorts on the basis of the design or analysis

a) ★ Study controls for the last menstrual period using qualified information of menstrual cycles.

b) ★ Study controls for the premature proportion and small for gestational age proportion between the early-ultrasound and last menstrual period references for gestational age, using statistics. c) There was no comparison for the premature proportion and small for gestational age proportion between the early-ultrasound and last menstrual period references for gestational age, using statistics.

***Outcome (3 stars):***

6) Assessment of outcome

a) ★ Independent blind assessment.

b) ★ Record linkage of database.

c) Zero: no description.

7) Statistical test and missing data

a) ★ The statistical test used to analyze the data is clearly described and appropriate, and the measurement of the association is presented, including confidence intervals and the probability level (p value).

b) ★ Missing data unlikely to introduce bias - small number lost <=10 %. c) Zero: Missing data >10% and no description of those lost.

d) Zero: No statement.

4
