# Supplementary material for: Influence of different methods for calculating gestational age at birth on prematurity and small for gestational age proportions: a systematic review with meta-analysis
Source: BMC Pregnancy Childbirth. 2023 Feb 11;23:106. doi: 10.1186/s12884-023-05411-0 (PMC9921121; doi:10.1186/s12884-023-05411-0)
Supplement: Supplementary file 2 — Additional file 2. Forest plot of premature proportions by last menstrual period and USG before 24 weeks without duplicated proportions [file 12884_2023_5411_MOESM2_ESM.docx]

**Additional file 2.** Forest plot of premature rates by last menstrual period and USG before 24 weeks without duplicated rates.


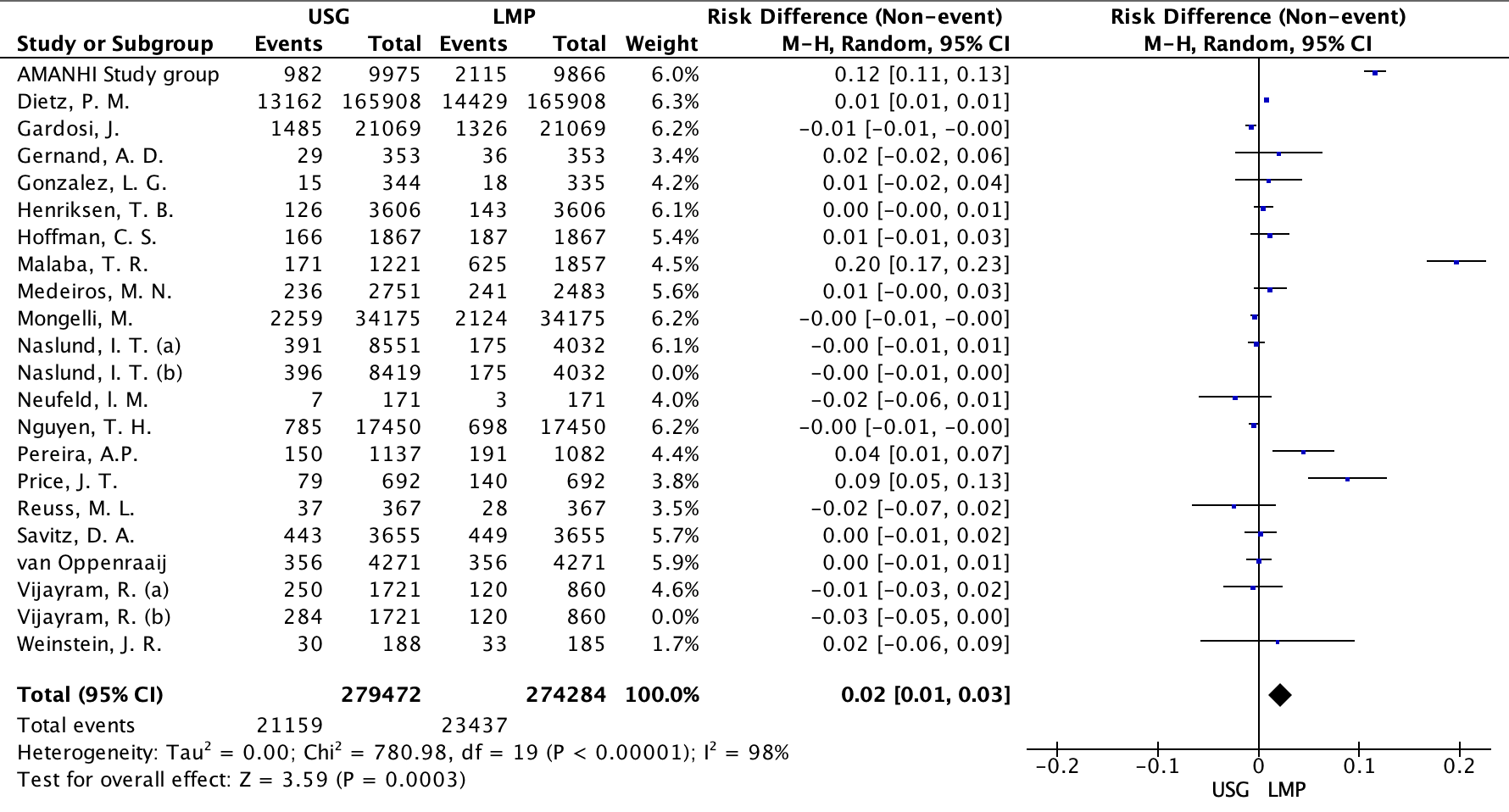


**Legends**: LMP: last menstrual period; USG: ultrasonography. Naslund (a): CRL measurement; Naslund (b): BPD measurement in the first trimester. Vijayram (a): USG Hadlock; Vijayram (b): USG Robinson-Fleming formula.
